# Spatial variability of urban forest topsoil properties: towards representative and robust sampling design

**DOI:** 10.12688/openreseurope.13502.2

**Published:** 2023-10-18

**Authors:** Nadina Galle, William Brinton, Robin Vos, Fábio Duarte, Marcus Collier, Carlo Ratti, Francesco Pilla

**Affiliations:** 1 Senseable City Laboratory, Massachusetts Institute of Technology, MIT 9-216, 77 Massachusetts Avenue, Cambridge, MA, 02139, USA; 2 Spatial Dynamics Lab, College of Engineering and Architecture, University College Dublin, Belfield, Dublin, D04 V1W8, Ireland; 3 Woods End Laboratories, 290 Belgrade Rd, Mt Vernon, ME, 04352, USA; 4 Commonland Foundation, Amsterdam, The Netherlands; 5 Pontifícia Universidade Católica do Paraná, Curitiba, Brazil; 6 School of Natural Sciences, Trinity College Dublin, Dublin 2, Ireland

**Keywords:** soil disturbance, spatial variability, soil quality, basal soil respiration, electrical conductivity, urban forestry

## Abstract

**Background:** Soil spatial variability is a major concern when deciding how to collect a representative topsoil sample for laboratory analysis. Sampling design to capture site-specific variability is documented in the agricultural literature, but poorly understood for urban forest soils where soils may be characterized by strong horizontal and vertical variability and large temporal anthropogenic disturbances.

**Methods:** This paper evaluates the spatial variability of selected topsoil properties under urban trees to define a statistically robust sampling design that optimizes the number of samples to reliably characterize basal soil respiration (BSR), a property associated with soil health. To provide a reference on variability, two additional soil properties were measured, unrelated to BSR: electrical conductivity (EC) and bulk density (BD). Thirteen sampling sites comprising both park and street trees ( *Acer rubrum*) were selected in Cambridge, MA, USA.

**Results:** Results indicate street tree topsoil had approximately twice as much variation, requiring more intensive sampling, as did park tree topsoil, even though street trees had smaller soil sampling zones, constricted by tree pits. The variability of BSR was nearly identical to that of EC, and BD results varied least. A large number of samples would be required for acceptable levels of statistical reliability (90% CI - 10% ER) of 44.4, 41.7, and 6.4 for BSR, EC, and BD, respectively, whereas by accepting a lower level of certainty (80% CI - 20% ER) the number of required soil samples was calculated as 6.8, 6.4, and 0.4 for BSR, EC, and BD, respectively.

**Conclusions:** The use of EC testing as a baseline measure to determine spatial variation in the topsoil is proposed, to alleviate the financial implications of more expensive BSR testing. Factors of topsoil disturbance and soil access restrictions at sites with severe root-sidewalk conflicts and the overall generalizability of the results are also discussed.

## Plain language summary

How many topsoil samples should I take from under this tree? That was the question we asked in this study.

On farms, where we've been taking soil samples for decades, there are guidelines to tell us how many samples we should take, but we do not have that information for the soil under urban trees. It is important to sample, test, and improve urban forest soil because healthy soil is imperative to grow big, mature trees in our cities.

To answer our research question, we took nine topsoil samples (0–15 cm) from under five street trees and seven park trees in Cambridge, MA, USA. Each topsoil sample was tested for three soil properties: basal soil respiration (BSR; the change in CO2 concentration over 24 hours), electrical conductivity (EC; the salinity), and pH (the acidity or basicity). Each subset of nine topsoil samples was compared per soil property. The closer the soil property values, the fewer topsoil samples you need for the site; the farther apart the soil property values, the more topsoil samples you need to achieve a representative and robust sample of that soil property for that tree.

The findings were surprising. Street tree topsoil had approximately twice as much variation, requiring more intensive sampling, as did park tree topsoil, even though street trees had smaller soil sampling zones, constricted by tree pits. We concluded the article by discussing the use of cost-effective EC testing to first determine how spatially variable the soil is, before pursuing more expensive BSR testing. The research was limited by only being able to sample topsoil (0–15 cm) due to severe soil compaction; simultaneously a limitation and reminder of how important it is to monitor and care for our urban forest soils.

## Introduction

The world’s population is moving to cities at a rate of three million people per week ( UN-Habitat, 2009; UN DESA, 2015), an unprecedented era of urban growth. Urban development typically results in immediate losses of vegetation ( Luck & Wu, 2002), after which, natural ecosystems are rapidly replaced by heterogeneous landscapes of impervious surfaces, buildings, and various types of green infrastructure. This process of urbanization is driving widespread loss of soil horizons, leaving only sparse patches of our planet’s “biologically active skin” strewn in between buildings and streets ( Wall *et al.*, 2010, p. 297), and subsequently, disrupting the basis for urban forest-based solutions ( Herrmann *et al.*, 2018).

The ecosystem services provided by urban forest-based solutions (e.g., biodiversity conservation, shading, carbon sequestration, and storage) will be constrained by the quality of urban soil. Urban soil quality has been poorly investigated due to the complexity of urban environments (e.g., soil cover, land-use history, pollution, degradation) ( Ferreira *et al.*, 2019). Yet, as local governments around the world choose to expand the quantity (and quality) of their urban canopies, the quality of this substrate will become increasingly pertinent ( Levin *et al.*, 2017; Pregitzer *et al.*, 2016).

Since industrialization, understanding urban soil density, moisture, and structure have been critical to geotechnical and civil engineering to support built infrastructures such as subways, sanitary systems, and the foundations of buildings ( Pincetl, 2010). Now, as more research demonstrates the importance of ecosystem services to the health and well-being of contemporary urban citizens, urban soils must be better understood, and their status more easily, rapidly assessed. There is a need to consider implications for ecosystem services derived from urban landscapes and to inform the transition to sustainable cities ( Herrmann *et al.*, 2018; Mitsch, 2012).

Soil health used interchangeably in this paper with soil quality, is defined as the capacity of soil to function as a vital living system within the ecosystem and land-use boundaries to sustain plant and animal productivity, maintain or enhance water and air quality, and promote plant and animal health ( Doran & Zeiss, 2000; Jemison *et al.*, 2019). In urban environments, anthropogenic influences (e.g., foot traffic, pollution, construction) have had such a ubiquitous and detrimental effect on soil health that they have given rise to an entirely new soil classification: anthrosol, from anthropogenic soil. The health of urban soils faces distinct challenges ( Tresch *et al.*, 2018), “as their evolution is controlled almost exclusively by humans, who impose very rapid transformation cycles compared with those occurring in less disturbed areas” ( De Kimpe & Morel, 2000, p. 31). As such, mechanical mixing of soil by plows or bulldozers, compaction of soil by machinery or trampling feet, and other anthropogenic environmental conditions can cause highly disturbed and variable soil ( Vasenev *et al.*, 2013).

When soil properties in urban soils are frequently characterized by strong horizontal and vertical variability ( Greinert, 2015; Pregitzer *et al.*, 2016), the number of samples taken when conducting soil testing is critical for achieving representative results. Urban forestry practitioners and scientists must justify the number of soil samples taken to ensure the analysis is robust for policy making, and so research committees can ensure the study is scientifically sound. Spatial variation in topsoil testing is well-documented in the agricultural literature, and best practices for soil sampling design have emerged over the last century ( Brinton *et al.*, 2019; Morton *et al.*, 2000; Parkin, 1993). However, due to strong soil property variability and spatial implications for testing, agricultural best practices do not apply to urban soils.

Soil variability characteristics in urban environments do not follow normal statistical distributions and most certainly not “regular spatial distributions”, referring to organized and predictable arrangements of soil properties across a study area. These arrangements exhibit consistency, predictability, and often take recognizable geometric forms. Their presence is statistically significant, aiding in the understanding of soil heterogeneity and the development of effective sampling strategies.

Field variability can have a five-times greater influence on soil variability than laboratory issues ( Brinton, 2020). Currently, the number of samples taken when testing soil health of urban forest-based solutions is based on feeling and intuition ( De Kimpe & Morel, 2000). A specific approach is needed to study such deeply disturbed environments in order to overcome the problems related to soil variability and representative soil testing. The original contribution of this paper is a robust, statistically derived soil sampling method for urban forest topsoil to optimize the number of samples depending on the topsoil disturbance, which varies greatly from street trees (defined as trees growing along public streets) to park trees (defined as trees in public parks). The study offers urban foresters and soil ecologists specific recommendations on the optimal number of samples required for basal soil respiration (BSR), electrical conductivity (EC), and bulk density (BD), three of the most commonly measured soil properties, and critical to urban tree health with direct relations to microbial activity, (excess) salinity, and compaction, respectively.

## Methods

### Location, tree selection, and topsoil sampling

**Figure 1.**
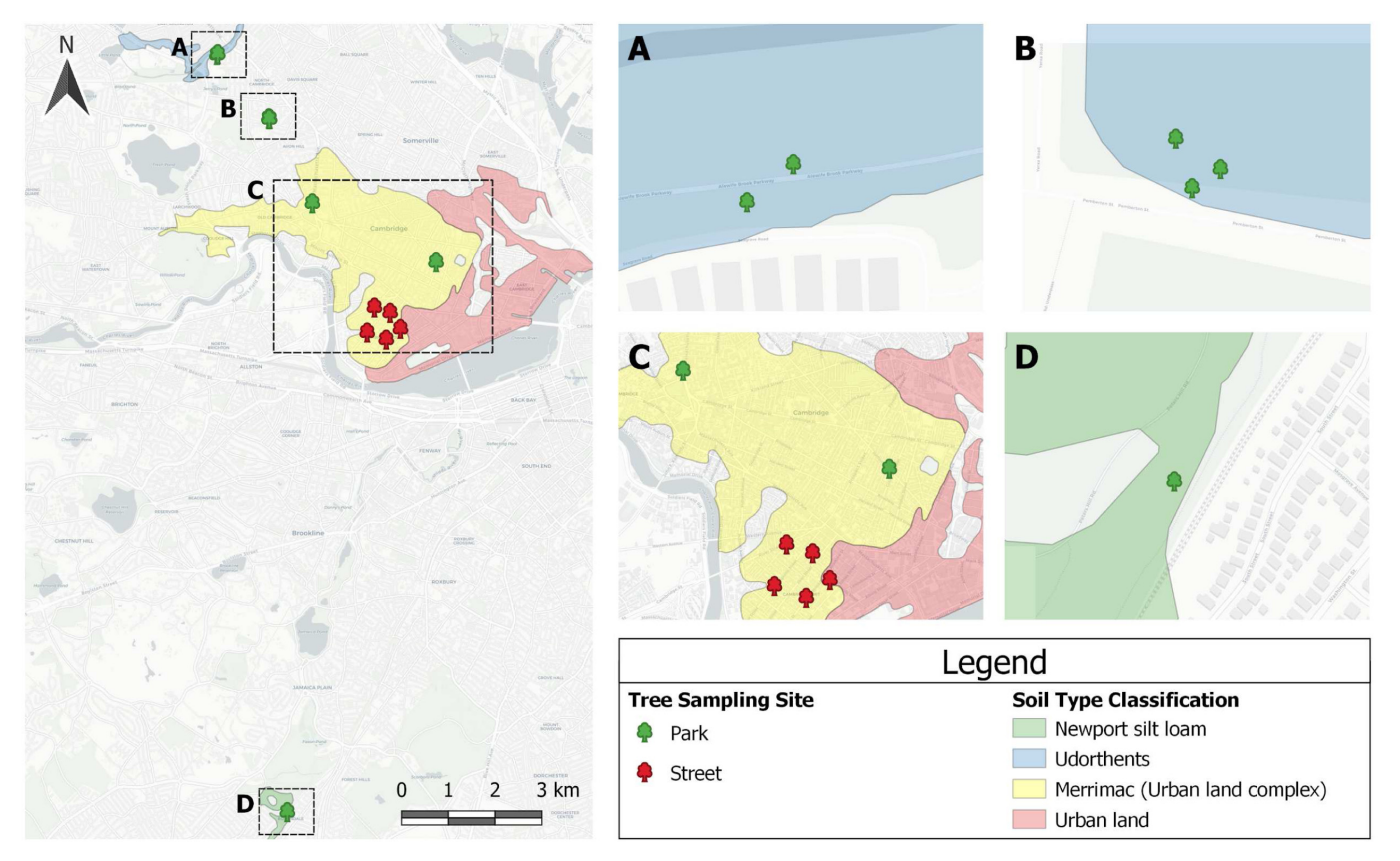
The tree sampling sites were widely distributed throughout Cambridge, with a control sample from Boston, MA. The majority (92%) of the sites were on urban soils, which are extensively influenced by human activities. Only the control sample had a unique soil type classification. This Soil Survey Geographic (SSURGO) database was produced by the U.S. Department of Agriculture (USDA), Natural Resources Conservation Service (NRCS), and cooperating agencies for the Soil Survey of Middlesex County, MA.

The soil study was carried out in Cambridge, Massachusetts, USA ( Figure 1). The City of Cambridge Department of Public Works granted permission to sample the topsoil as required. Cambridge is a medium-sized city with 118,977 people, located in the state of Massachusetts and part of the Boston metropolitan area. The city’s history dates back to the beginning of the seventeenth century. Mining the Alewife Brook subwatershed for clays (to sustain Boston’s early brick industry), and the development that followed, caused extensive disturbance to Cambridge’s surface soils. Cut and fill activities left most of Cambridge with anthropogenically-influenced soils, which now lack properties of naturally developed soils, such as defined layers and horizons, permeability, and microbial activity ( Cambridge Department of Public Works, 2008). These conditions act as a poor substrate for the city’s urban forestry and other greening initiatives.

Genetically, all tree roots are capable of growing several meters in depth. However, most urban soils, which are typically compacted and have poor drainage, most often result in shallow rooted trees. Cambridge’s heavily anthropogenically-influenced soil have made shallow rooted trees the rule, rather than the exception. Therefore, soil samples were taken to a depth of 15 cm. At most sites, the soil we had access to was so compacted it prevented the corer from deeper sampling.

That being said, in agriculture, a 0–15 cm soil sampling depth is normally sufficient, but there is limited literature on urban forest soil sampling. In some similar studies, like Moriyama & Numata (2015), when investigating cicada diversity loss, the authors sampled severely compacted urban soil to a maximum depth of only 5 cm, affirming shallow sampling for surface soil compaction. In another citywide analysis of 136 urban sites, sampling was limited to 14 cm, also due to severe compaction ( Edmondson *et al.*, 2011). Since total rooting depth is unknown, the topsoil was severely compacted, and previous literature to support a shallow soil sampling depth, the decision was made to sample 0–15 cm, or the “topsoil”.

A total of 117 soil samples were collected to sample the topsoil of thirteen red maples ( *Acer rubrum*), including five street trees, seven park trees, and one reference sample from Boston’s Arnold Arboretum (Section D, Figure 1). The reference, an unmodified topsoil, was collected from a “no-mow area” within Peter’s Hill at the arboretum. The reference sample was identified on SSURGO (Soil Survey Geographic Database) as a coarse-loamy, mixed, mesic Newport silt loam, Typic Dystrochrept, soil order Inceptisol. For all other soils, the man-made nature of the sites suggested they be loosely classified as, “soils with strong human influence”, including Anthrosols (i.e., soils exposed to continuous and intensive agriculture) and Technosols (i.e., soils impacted by industrial interventions), of unknown origin ( International Union of Soil Sciences Working Group, 2015, p. 10). The USDA Soil Taxonomy (2010) refers to these anthropogenic soils as Udorthents and (one of) its suborders: Merrimac urban land complex. 

 The red maple was chosen because maples dominate Cambridge’s landscape, as they do other North American urban landscapes, growing as far south as Florida and as far north as Quebec (Canada). Its geographical spread offers ideal preconditions for this study’s replicability. While it prefers wet conditions (and even tolerates standing water) and acidic soils, the red maple is highly adaptable to less-than-ideal conditions, making it a true survivor, especially in the urban context.

Nine samples were taken from each of the 13 sites for a total of 117 topsoil samples. Topsoils were sampled from October 14–21, 2019, with a 15 cm long Ames hand bulb planter (10 cm wide) to a depth of 15 cm. A more sophisticated soil corer was also attempted (brand name: JMC “Backsaver Handle”) but at most sites, soil contained obstructions such as roots and infrastructure that prevented the corer from sampling deeper than 15 cm. The bulb planter also gave sufficient volume per sample, which minimized time spent and disturbance to already vulnerable topsoil. The weather conditions on all sampling days varied from sunny to partly cloudy, with temperatures ranging from 12 to 18°C. No samples were taken within a 48-hour period of a precipitation event to ensure proper soil moisture range ( Brinton, 2019; Salter & Williams, 1965). It had rained for two days 72 hours prior to the first sampling date. It did not rain between the sampling dates (October 14–21, 2019).

**Figure 2.**
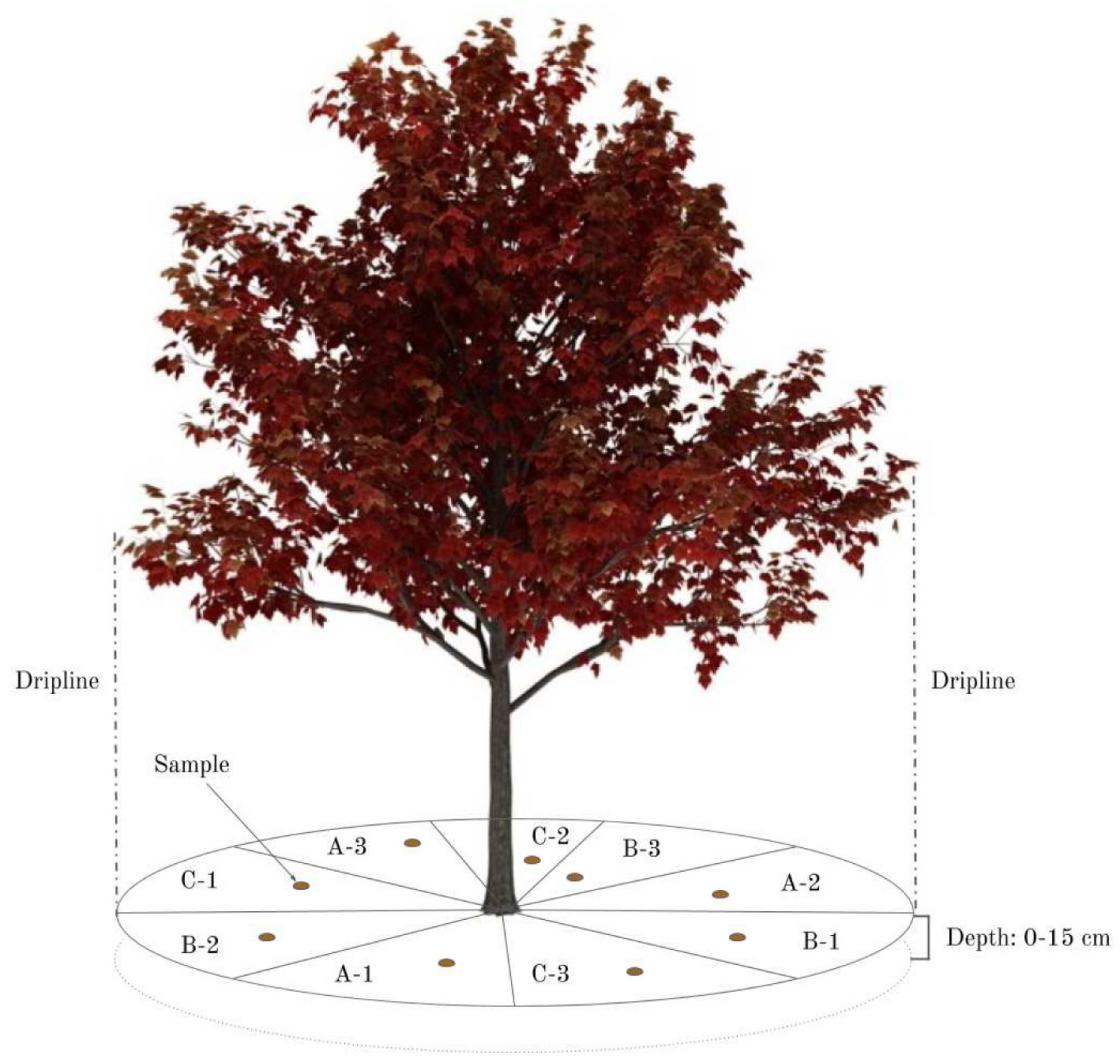
Sampling design within the tree’s dripline, divided into nine equal parts. The hypothesis is that any three of these zones will accurately represent the soil under the tree.

Surface vegetation, debris, and humus were removed with a trowel. Within each tree’s dripline (i.e., the outermost circumference of the tree's canopy), the soil was divided into nine equal zones ( Figure 2: A-1; A-2; A-3; B-1; B-2; B-3; C-1; C-2; C-3). For street trees, these zones became smaller, as there was a limited soil access area. Each zone, and each topsoil core within each zone, was selected at random for each individual tree sampled ( Figure 2). It was not possible to note the GPS-coordinates of each topsoil core, as the area was too small and the GPS-variability too high. The GPS-coordinates for each tree sampled were recorded at the trunk.

 Then, one 15 cm topsoil sample was taken within each zone and collected into labelled, sealable plastic bags. The samples were rapidly transported back to the testing location to prevent sun exposure. New England autumn temperatures (12 to 18°C) helped keep soils cool.

### Topsoil testing and analytical methods

The standard operating protocol for the Solvita Field Test was followed ( Brinton, 2018) to measure SBR. Samples were sieved through a 6 mm sieve to remove roots, leaf litter, and/or gravel. A digital scale (0.1 g readability) was used to weigh 90-gram topsoil samples, which were labelled, and placed into polystyrene incubation jars (475 ml) with gas-tight gasket lids. The topsoils were tested on an as-is moisture basis. One low-level CO _2 _gel probe was added. Samples were placed in a dark room at room temperature (22°C). CO _2 _gel probes were read after 24 hours and recorded with the Solvita Multi-Mode Digital Color Reader using ALT mode for BSR. Method variance testing was conducted as a QA/QC procedure with standard colour chips to ensure the Solvita Field Test was a reliable method for quantifying SBR.

EC was measured on a 1:1 soil:deionized (DI) water ratio by vortex-spinning for 10 seconds and then standing for 30 minutes before insertion of a flow-through EC probe with a cell constant of 10cm ^-1^ calibrated against a commercial KCl standard. Results were read to the nearest 0.01 dS/m ^-1^. BD was measured as g cc ^-1^ by placing dry, 2 mm sieved topsoil levelled in a standard, certified 30 cc scoop, and weighing to the nearest 0.1 gram.

The Snedecor & Cochran (1980) statistical methods were used to determine the required number of samples to achieve the desired certainty result. Two confidence intervals (CI) of 90% and 80% with an allowable error rate (ER) of 10% and 20% of the sample mean, were selected. The following formula by Brinton *et al.* (2019) was used:

$$n={\left(\frac{Z-S}{E}\right)}^{2}$$

*Where* *n* = sample size required

*z* = z score associated with the degree of confidence
*s* = sample standard deviation
*E* = allowable error, or half the width of the CI

For each tree sampled, the required number of samples to achieve 90% CI - 10% ER, 80% CI - 10% ER, 90% CI - 20% ER, and 80% CI - 20% ER was plotted for BSR, EC, and BD. All statistical analyses were conducted using Minitab statistical software (State College PA). In the paper, selected statistical figures and correlations are shown.

##  Results

All soil properties within the 0–15 cm depth interval for the 117 topsoils sampled are presented in the *Underlying data* ( Galle *et al.*, 2021). The number of samples required at 90% CI - 10% ER and 80% CI - 20% ER for BSR, EC, and BD for both park and street trees are presented below. The number of samples required at the other CIs and ERs, 80% CI - 10% ER, and 90% CI - 20% ER, can be found in the *Underlying data* ( Galle *et al.*, 2021).

pH measurements were conducted on a total of 24 soil samples collected from locations in Boston and Cambridge, both of which are closely situated within a 3 km radius. The average pH value obtained from these samples was determined to be 5.61 ± 0.59. This pH level indicates that the soils in the studied areas can be characterized as moderately acidic. Importantly, this pH range does not suggest any significant deviation from the initially anticipated values, suggesting relative stability in soil acidity levels over the study period.

### Site characteristics

SBR (mg/kg CO _2_-C) varied widely from site to site, and within sites. Values as low as 7.4 ± 2.256 mg/kg CO _2_-C (mean ±1 SE) were observed at AR8 (park), and values as high as 138 ± 2.256 mg/kg CO _2_-C at AR10 (street). Three samples (AR7-B2; AR10-A3; AR11-C1) were removed as outliers because they were ≥125 mg/kg CO _2_-C, which is considered excessively high, according to the Solvita Field Test interpretation key. AR7 was a park tree, AR10 and AR11 were street trees. Electrical conductivity varied somewhat but not significantly less. The lowest value was 0.08 ± 0.04 dS/m ^2^ at AR8 (park) and the highest was 2.34 ± 0.04 dS/m ^2^ at AR2 (street). Bulk density (g/cc) varied less. The lowest value was 13.6 ± 0.5 g/cc at AR11 (street) and the highest was 43.1 ± 0.5 g/cc AR3 (street).

### Number of samples required

For each topsoil property measured, the number of samples required at given CIs and ERs varied greatly. BSR showed the most variation with 44.4 samples required at 90% CI - 10% ER and 6.8 samples required at 80% CI - 20% ER. On average, street trees required nearly twice (± 1.76) as many samples as park trees, despite being constricted by tree pits and having much smaller soil sampling zones. This was true across all CIs ( Table 1 and Table 2).

**Table 1.** Mean number of samples required at a 90% and 80% confidence interval (CI) and 10% and 20% error rate (ER; % of mean) for basal soil replication (BSR) for park trees.

				Basal Soil Respiration			
				*Samples required at given CI and ER*			
				90% CI - 10% ER		80% CI - 20% ER	
Location	Sub group	BSR (mg/kg)	SD	Per sub group	Average per site	Per sub group	Average per site
C - park	A	37.6	4.86	4.5	2.7	0.7	0.4
	B	34.1	3.15	2.3		0.3	
	C	31.2	2.08	1.2		0.2	
AR1 - park	A	49.3	13.98	22	22	3.3	3.3
	B	42.2	5.45	4.5		0.7	
	C	39.5	15.14	39		6.0	
AR4 - park	A	32.3	2.21	1.3	4.1	0.2	0.6
	B	29.2	4.73	7.0		1.1	
	C	28.0	3.39	3.9		0.6	
AR5 - park	A	13.4	2.50	9.3	32	1.4	4.8
	B	33.3	17.77	77		12	
	C	15.1	2.71	8.6		1.3	
AR6 - park	A	20.9	10.15	63	57	9.7	8.6
	B	16.7	4.35	18		2.8	
	C	16.3	9.32	88		13	
AR7 - park	A	45.4	12.92	22	31	3.3	4.7
	B	33.1	5.87	8.5		1.3	
	C	26.7	12.95	63		9.6	
AR8 - park	A	16.3	8.30	70	52	11	8.0
	B	17.6	8.89	69		10	
	C	22.7	5.85	18		2.7	
AR9 - park	A	72.9	24.04	29	74	4.5	11
	B	18.4	2.62	5.4		0.8	
	C	56.5	47.27	188		29	

**Table 2.** Mean number of samples required at a 90% and 80% confidence interval (CI) and 10% and 20% error rate (ER; % of mean) for basal soil replication (BSR) for street trees.

				Basal Soil Respiration			
				*Samples required at given CI and ER*			
				90% CI - 10% ER		80% CI - 20% ER	
Location	Sub group	BSR (mg/kg)	SD	Per sub group	Average per site	Per sub group	Average per site
AR2 - street	A	28.9	4.83	7.5	16	1.1	2.5
	B	27.3	6.42	15		2.3	
	C	37.9	12.00	27		4.1	
AR3 - street	A	48.8	46.92	248	207	38	32
	B	46.8	46.00	260		40	
	C	37.3	24.11	113		17	
AR10 - street	A	21.0	5.30	17	41	2.6	6.2
	B	38.6	2.70	1.3		0.2	
	C	25.8	15.97	103		16	
AR11 - street	A	38.8	12.08	26	11	4.0	1.7
	B	33.0	1.15	0.3		0.1	
	C	33.6	5.16	6.4		1.0	
AR12 - street	A	14.0	2.37	7.7	27	1.2	4.2
	B	18.6	4.91	19		2.9	
	C	19.7	8.96	56		8.5	

EC had very similar results to BSR, with 41.7 samples required at 90% CI - 10% ER and 6.4 samples required at 80% CI - 20% ER. Like BSR, street trees also required nearly twice (± 1.65) as many samples as park trees ( Table 3 and Table 4).

**Table 3.** Mean number of samples required at a 90% and 80% confidence interval (CI) and 10% and 20% error rate (ER; % of mean) for electrical conductivity (EC) for park trees.

				Electrical Conductivity			
				*Samples required at given CI and ER*			
				90% CI - 10% ER		80% CI - 20% ER	
Location	Sub group	EC (dS/m2)	SD	Per sub group	Average per site	Per sub group	Average per site
C - park	A	0.32	0.21	114	41	17	6.2
	B	0.25	0.04	6.3		1.0	
	C	0.17	0.02	2.1		0.3	
AR4 - park	A	0.47	0.10	12	20	1.8	3.1
	B	0.48	0.07	5.7		0.9	
	C	0.68	0.27	43		6.5	
AR5 - park	A	0.39	0.22	84	34	13	5.2
	B	0.69	0.17	16		2.4	
	C	0.24	0.02	2.1		0.3	
AR6 - park	A	0.34	0.08	16	27	2.5	4.0
	B	0.29	0.10	30		4.5	
	C	0.26	0.09	33		5.1	
AR7 - park	A	0.27	0.16	95	37	14	5.7
	B	0.23	0.05	14		2.2	
	C	0.18	0.02	3.3		0.5	
AR8 - park	A	0.10	0.02	8.1	19	1.2	2.9
	B	0.13	0.05	34		5.1	
	C	0.13	0.03	16		2.4	
AR9 - park	A	0.36	0.15	46	52	7.0	7.9
	B	0.29	0.17	91		14	
	C	0.32	0.09	19		2.8	

**Table 4.** Mean number of samples required at a 90% and 80% confidence interval (CI) and 10% and 20% error rate (ER; % of mean) for electrical conductivity (EC) for street trees.

				Electrical Conductivity			
				*Samples required at given CI and ER*			
				90% CI - 10% ER		80% CI - 20% ER	
Location	Sub group	EC (dS/m2)	SD	Per sub group	Average per site	Per sub group	Average per site
AR2 - street	A	0.65	0.14	12	75	1.8	11.5
	B	1.20	1.00	186		28	
	C	0.61	0.20	28		4.3	
AR3 - street	A	0.71	0.61	198	98	30	15
	B	0.96	0.51	77		12	
	C	1.32	0.34	18		2.7	
AR10 - street	A	1.20	0.75	105	68	16	10.3
	B	1.00	0.16	7.3		1.1	
	C	0.98	0.75	92		14	
AR11 - street	A	0.28	0.05	7.1	7	1.1	1.0
	B	0.29	0.04	5.3		0.8	
	C	0.22	0.04	7.1		1.1	
AR12 - street	A	0.15	0.05	28	23	4.2	3.6
	B	0.17	0.05	23		3.5	
	C	0.15	0.04	19		2.9	

The number of samples required for BD was drastically less than BSR and EC, with 2.4 samples required at 90% CI - 10% ER and 0.4 samples required at 80% CI - 20% ER. Significant spatial variation in street trees compared to park trees was concluded; street trees required nearly five times (± 4.89) as many samples as park trees ( Table 5 and Table 6).

**Table 5.** Mean number of samples required at a 90% and 80% confidence interval (CI) and 10% and 20% error rate (ER; % of mean) for bulk density (BD) for park trees.

				Bulk Density			
				*Samples required at given CI and ER*			
				90% CI - 10% ER		80% CI - 20% ER	
Location	Sub group	BD (g/cc)	SD	Per sub group	Average per site	Per sub group	Average per site
C - park	A	0.91	0.03	0.2	0.4	0.0	0.1
	B	0.86	0.02	0.2		0.0	
	C	0.92	0.05	0.9		0.1	
AR4 - park	A	0.98	0.04	0.5	0.4	0.1	0.1
	B	0.95	0.03	0.2		0.0	
	C	1.03	0.04	0.4		0.1	
AR5 - park	A	1.01	0.04	0.4	1.6	0.1	0.2
	B	1.01	0.12	3.6		0.6	
	C	1.17	0.06	0.6		0.1	
AR6 - park	A	1.07	0.10	2.6	1.2	0.4	0.2
	B	1.00	0.05	0.6		0.1	
	C	1.03	0.04	0.5		0.1	
AR7 - park	A	0.96	0.03	0.4	0.4	0.1	0.1
	B	0.97	0.05	0.6		0.1	
	C	1.03	0.02	0.1		0.0	
AR8 - park	A	1.19	0.07	1.0	1.1	0.2	0.2
	B	1.12	0.10	2.1		0.3	
	C	1.18	0.03	0.2		0.0	
AR9 - park	A	1.05	0.10	2.6	2.0	0.4	0.2
	B	1.21	0.03	2.1		0.0	
	C	1.02	0.07	1.4		0.2	

**Table 6.** Mean number of samples required at a 90% and 80% confidence interval (CI) and 10% and 20% error rate (ER; % of mean) for bulk density (BD) for street trees.

				Bulk Density			
				*Samples required at given CI and ER*			
				90% CI - 10% ER		80% CI - 20% ER	
Location	Sub group	BD (g/cc)	SD	Per sub group	Average per site	Per sub group	Average per site
AR2 - street	A	1.18	0.10	2.1	2	0.3	0.3
	B	1.13	0.12	3.1		0.5	
	C	1.23	0.02	0.1		0.0	
AR3 - street	A	1.30	0.06	0.7	3	0.1	1
	B	1.21	0.17	5.5		0.8	
	C	1.28	0.15	3.8		0.6	
AR10 - street	A	0.97	0.13	4.7	4	0.7	0.6
	B	0.87	0.10	3.8		0.6	
	C	1.15	0.13	3.3		0.5	
AR11 - street	A	0.70	0.06	2.1	13	0.3	1.9
	B	0.53	0.06	4.0		0.6	
	C	0.69	0.24	32		4.9	
AR12 - street	A	1.14	0.05	0.5	1	0.1	0.1
	B	1.11	0.05	0.6		0.1	
	C	1.12	0.08	1.3		0.2	

The number of samples required at the other CIs and ERs, 80% CI - 10% ER and 90% CI - 20% ER, can be found in the *Underlying data* ( Galle *et al.*, 2021).

### Non-normal data

Spatial variation across BSR, EC, and BD did not follow normal distribution patterns and most certainly not regular spatial distributions. For correlating basal respiration to EC outlier separations were first used to better identify the spatial groupings and then Box-Cox transformation applied to all samples together. To correlate BSR with BD a Johnson transformation was required for BD to be used successfully.

**Figure 3.**
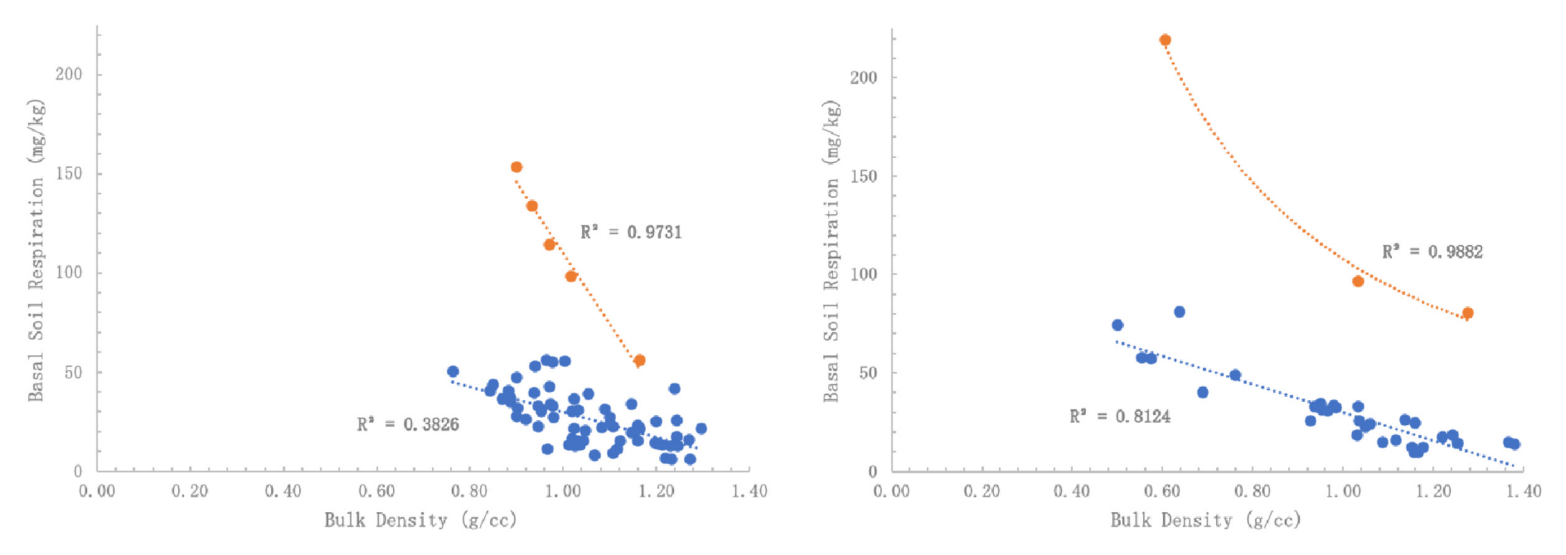
( **a**) Basal soil replication (BSR) of park trees as affected by bulk density (BD) of the regular group (R ^2^ = 0.3826) versus high respiration outlier group (R ^2^ = 0.9731) and ( **b**) BSR of street trees as affected by BD of the regular group (R ^2^ = 0.8124) versus high respiration outlier group (R ^2^ = 0.9882). The apparent relationship indicates that stronger respiration is due to increased organic matter associated with lower BD soils.

BSR versus BD showed a typical grouping. Outliers were separated, which revealed predictable distribution. There appears to be a relationship of BD to the BSR rate ( Figure 3). This may mean lower BD topsoil have stronger respiration, likely due to increased organic matter causing lower densities. The BSR outlier topsoil appears to be separate populations and all correlations are significant. If normalized data is used (Box-Cox and Johnson) and all samples for the park and street trees are regressed together, a similar conclusion of BD influencing BSR rate is reached and is statistically highly significant (p < 0.001).

**Figure 4.**
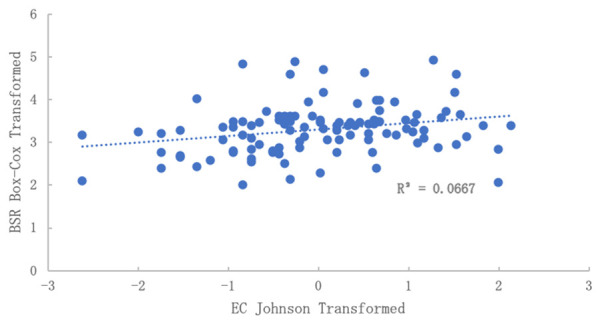
Relationship of electrical conductivity (EC) to basal soil replication (BSR) after normalization. No correlation was observed in the data before normalization. BSR was Box-Cox transformed and EC was Johnson transformed.

 The relationship between EC to BSR was also examined ( Figure 4). Using the original data, no correlation was observable. However, when applying Box-Cox transformed BSR and Johnson transformed EC, a very significant correlation was obtained, although the R ^2^ was low. Therefore, there is some reason to believe a secondary factor influencing BSR is the presence of dissolved salts, an indirect indicator of nutrient fertility.

## Discussion and further research avenues

Little work has explored the number of samples required for soil testing in urban forest topsoil. One similar study ( Cohen *et al.*, 2008) found that, for soil pH, three samples are needed, while more (n = 11–33) are needed for soil organic matter (SOM) and total phosphorus (TP). Cohen *et al.* (2008) assumed a 10% allowable error (% of mean), indicating that results are of similar statistical allowance. The Snedecor & Cochran (1980) statistical techniques shown in Cohen *et al.* (2008) mirrored that of this study (p. 419). Another study ( Conant & Paustian, 2002) also showed similar spatial variability estimates with comparable techniques (p. 131), but then detected changes in soil C in grasslands. So far, the only publication using this particular spatial variability model for BSR, EC, and BD was Brinton *et al.* (2019), which inspired this study.

Some of the very large variability observed in the BSR data may have been caused by the hydrophobic topsoil properties. Previous work found that urban forest topsoil was characterized by a significant accumulation of high molecular weight Polycyclic Aromatic Hydrocarbons (PAHs) of industrial and vehicular origin, likely due to urban industry and traffic. A semi-quantitative test of hydrophobicity was used and is the relative speed of wetting after the addition of water to dry soil. A soil that wets within 1 minute of water addition (9 cc added to 30 cc soil) would be considered normal; a soil that requires 10 minutes to wet is restricted, and a soil requiring 24 hours is considered extremely hydrophobic.

This paper’s results corroborate the findings of Blume (1986) and Craul & Klein (1980) that urban topsoil typically show great variability horizontally across the landscape. Craul (1991) concurs that this is, “primarily due to the cut and fill, backfilling, and resurfacing that occur during the process of land shaping” (p. 25). Results show more topsoil disturbance (e.g., in street trees) would cause a greater spatial variance, and therefore require more samples to gain an accurate and representative estimation of the topsoil’s properties. However, the association between smaller soil sampling zones and reduced spatial variance was disproven. Results suggest spatial variance increases as the soil sampling zone size decreases (e.g., tree pit). However, size is most likely not the only contributing factor. Sites with small soil sampling zones are highly likely to be street tree sites, which mostly have poorer quality topsoil. Findings from similar studies indicate that urban soils, especially underneath street trees, are highly compacted ( Craul, 1991). While tree roots are highly opportunistic and will grow wherever the environment is satisfactory, compaction will jeopardize that growth. Foot traffic, construction, and vehicles close pore space, reduce aeration and restrict absorption of water and oxygen ( Day & Bassuk, 1994). Ultimately, roots will not be able to penetrate the soil and the tree’s growth will be stunted.

**Figure 5.**
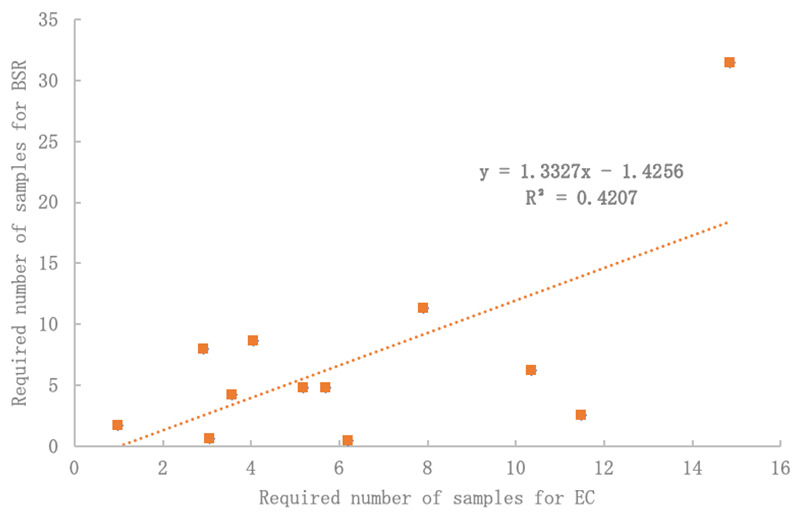
Electrical conductivity (EC) may be a suitable proxy for basal soil replication (BSR) variability with significant R ^2^ and no difference by Student’s T-Test (p < 0.05).

This study was focused on limited parameters. It tested (and ultimately advised) a topsoil sampling design based initially on BSR and then compared to EC and BD. These topsoil properties were chosen because they are three of the most commonly measured topsoil properties, and critical to urban tree health with direct relations to microbial activity, (excess) salinity, and compaction, respectively. In general, these properties are also indicative of overall topsoil health, methodically consistent, easy-to-measure, and had little between-sample method variance. Observing EC at the same locations as BSR expressed similar variation (r2 = 0.4207 p < 0.05) and was not statistically different from variation in BSR ( Figure 5). Therefore, controlling for at least one source of variability is likely to improve sampling design for all other types of analysis. Future research should explore this.

This study provides valuable insights into soil properties at our urban pilot site; however, it is essential to acknowledge certain limitations, particularly in the measurement of EC and BD.

Regarding BD measurement, our laboratory protocol, while consistently applied, tends to underestimate field BD. This discrepancy may be attributed to the inherent limitations of the laboratory method, which may not fully capture undisturbed BD in the field. This choice was necessitated by the urban pilot site's constraints, precluding the collection of deeper undisturbed soil samples, typically required for conventional BD measurements.

In our BD determination, we employed a soil transfer method, sieving dry soil into a 30cc scoop before measurement. This approach aimed to reflect real-world conditions, acknowledging potential variations in density during the scooping process.

These limitations, particularly in EC and BD measurements, should be considered when interpreting our results. While we ensured methodological consistency and accuracy, the urban pilot site's unique characteristics required deviations from standard protocols. Future research in similar constrained environments should explore alternative methods to enhance soil health evaluations.

Critical concerns remain over the cost-effectiveness of statistically robust sampling. The choice of sampling strategy may lie in its financial implications. This paper shows that several samples (or sensors) are required to achieve an accurate and representative estimation of topsoil properties in highly disturbed and variable urban environments. The choice of acceptable strategy may be dependent on considering the test cost due to the large number of samples indicated at any level of certainty. A sampling at 90% CI - 10% ER would appear unrealistic, and financially infeasible. We suggest accepting a 20% ER at either 90% or 80% CI for optimal sampling that still achieves accuracy but lessens costs.

In addition, before embarking on costly biological testing (like BSR), we propose using cheaper EC testing as a proxy to test for topsoil spatial variation. This approach is justified by our findings, which indicated that the range of EC values fell within the typical range for soils, revealing no indications of salt contamination. It is important to note that our sampling was conducted during the fall season, allowing a full year to elapse since the last potential salting activity, a practice minimally employed by city officials. Furthermore, our investigation did not reveal any discernible impact of "waste cleaning" water on the soil, and in cases of dog urine presence, it is reasonable to assume that such occurrences would be randomly distributed across the city. Considering the natural dispersion mechanisms associated with normal rainfall patterns, any potential influences on EC values from these sources would likely be mitigated. In light of these findings, the use of EC as a proxy for topsoil spatial variation is both justified and cost-effective for our study. Figure 5 shows significant comparisons between the required number of samples for EC and BSR. This was true across all CIs. Also, when normalizing data for both EC and BSR ( Figure 4), a significant correlation was found, which could indicate a strong relationship between changes in EC and biological response, respectively.

Another limitation was the severe root-sidewalk conflicts faced in the field. Initially, some 100 sampling locations were pre-selected based on the City of Cambridge tree inventory. However, nearly 70% of the street trees initially chosen were unable to be sampled due to insufficient soil volumes in the unrestricted area around the tree. It is likely that these trees, despite being alive, are in poor health due to a lack of growing matrix. To understand the spatial variance of these soils under severe "anthropedogenesis" ( Effland & Pouyat, 1997), also known as the role of human activity in soil formation, deep, continuous mechanical operations would be required to access the soil. We do not know if this kind of more intensive sampling would cause the tree more harm than good, but further studies should take the spatial variance of these inaccessible soils into account to also measure vertical variability.

This study only analyzed horizontal variability due to severe topsoil compaction uncovered across all sites, which hindered sampling deeper than 15 cm. Since only highly intrusive mechanical sampling could access deeper soils, involving possible and unknown damage to the trees, it was not possible to map vertical variation. There is, however, a reason to believe vertical soil profiles are more heterogeneous than we think. Herrmann *et al.* (2018) compared urban to reference pre-urban soil profiles from 11 different cities, which revealed how urbanization modifies the presence and order of soil layers and its properties. They found urban soils had fewer horizons than their pre-urban counterparts, and often missed intermediate B horizons, which are critical to certain ecosystem functions. Future research should examine the role of widespread loss of intermediate soil horizons on vertical variation in soil properties, and ultimately, its impact on urban tree health.

Studies continue to show urban soils are becoming increasingly heterogeneous ( Effland & Pouyat, 1997; Hope *et al.*, 2005; Pouyat & McDonnell, 1991). However, urbanization processes (e.g., cut and fill, backfilling) are increasingly similar worldwide, which may support a generalized understanding of the nature of urban topsoil on a global scale. While future research should also consider the applicability of this study’s results to other cities, we may be approaching an era where urban soils are increasingly homogeneous. The ‘next generation’ soil sampling design and common metrics proposed in this study could indeed be widely employed.

## Conclusions

An increased interest in topsoil quality as it relates to urban tree health and growth raises important challenges in how measurements are to be made in documenting topsoil health. In this study, horizontal spatial variation of topsoil quality traits including a biological indicator (BSR), EC, and BD was measured using 117 topsoil samples across 13 tree sites to recommend an urban forest topsoil sampling design for practitioners. The results underscore the challenge to obtain a robust, statistically derived topsoil sampling method to accommodate the observed topsoil-based variability associated with the urban forest. Among key findings, the study revealed that topsoil underneath street trees had twice as much variation as did topsoil under park trees, although street trees were constricted by tree pits and had much smaller soil sampling zones.

Human-induced activities such as construction and landscaping clearly increase potential variability in an urban measurement environment, and additionally at the street-level, depositions including litter and vehicular hydrocarbon output result in increased perturbations affecting test results. In the urban environment, trees must survive severe root-sidewalk conflicts, and these translate to various impediments for topsoil sampling. These factors taken as a whole suggest that before any conclusions on tree health as affected by topsoil quality can be made, a statistically validated sampling regimen should be derived that constrains sample variability to acceptable limits. These data suggest a number of directions that urban foresters, scientists, and practitioners should explore in topsoil sampling as a means to gain a better understanding of tree health.

Urban soil science is an emerging field of interest. Urban environments are increasingly becoming humans’ dominant ecosystem, as approximately three million people move to cities every week. Growing urban biomes bring about a unique set of challenges for keeping cities clean, comfortable, and healthy places to live. Healthy soil, with ample microbes, underlies the urban forest-based solutions to several of those challenges, yet is fast becoming a limited commodity in cities. Geotechnical and civil engineers analyze soil as a substrate for laying the foundation of the “built environment”. If, however, the lens of ecological engineering is applied, it may influence decisions about sustainable ecosystems that integrate human society with its environment. While soil is intrinsically linked with anthropogenic activities in cities, it is also essential for the functioning of urban ecosystems. Transitioning to urban forest-based cities will potentially require that ecological engineers and related professionals consider the implications of proper topsoil sampling methodology to characterize a soil’s ecosystem services in the cities of tomorrow.

## Data Availability

Zenodo: Mean number of samples needed at a 90% and 80% CI and 10% and 20% ER (% of mean) for BSR, EC, and BD for park trees and street trees. https://doi.org/10.5281/zenodo.4687540 ( Galle *et al.*, 2021). Data are available under the terms of the Creative Commons Attribution 4.0 International license (CC-BY 4.0).
