# Supplementary material for: Spatial variability of urban forest topsoil properties: towards representative and robust sampling design
Source: Open Res Eur. 2023 Oct 18;1:45. Originally published 2021 Apr 29. [Version 2] doi: 10.12688/openreseurope.13502.2 (PMC10587662; doi:10.12688/openreseurope.13502.2)
Supplement: Peer review history

# Reviewer report: Reviewer response for version 2

**DOI:** 10.21956/openreseurope.18020.r35692

**Published:** 2023-10-20

**Authors:** Nina Bassuk

**Affiliations:** 1 Urban Horticulture Institute, Cornell University, Ithaca, NY, USA

I approve the revised version of this manuscript.

Is the study design appropriate and does the work have academic merit?

Partly

Is the work clearly and accurately presented and does it cite the current literature?

Yes

If applicable, is the statistical analysis and its interpretation appropriate?

Yes

Are all the source data underlying the results available to ensure full reproducibility?

Partly

Are the conclusions drawn adequately supported by the results?

Partly

Are sufficient details of methods and analysis provided to allow replication by others?

No

Reviewer Expertise:

Urban soil and plant selection. Site assessment and soil modification

I confirm that I have read this submission and believe that I have an appropriate level of expertise to confirm that it is of an acceptable scientific standard.

# Reviewer report: Reviewer response for version 1

**DOI:** 10.21956/openreseurope.14572.r28917

**Published:** 2022-04-04

**Authors:** Nina Bassuk

**Affiliations:** 1 Urban Horticulture Institute, Cornell University, Ithaca, NY, USA

I have some questions relating to the methods of determining bulk density. It appears that dry soil was sieved and then placed into a 30cc scoop before weighing. This is not an undisturbed soil sample. Error of packing into the scoop would necessarily affect the density and thus the weight of the soil. This method is not standard for undisturbed bulk density.

 Also, in the abstract,  you mention that pH was determined, yet no results of soil pH were reported. This would be interesting because Cambridge generally has an acidic soil yet anthropogenic activities might change the soil pH. An opportunity lost.

 By concluding that EC is a proxy for BSR, there is no acknowledgement that anthropogenic salts( de-icing salt, waste cleaning water, dog urine) might affect EC.

 In conclusion, the number of samples may be adequate but the methodology concerning EC and bulk density is questionable. Because the methods can be compromised due to anthropogenic factors affecting EC or the BD measurement technique, it would be difficult to replicate the results.

Is the study design appropriate and does the work have academic merit?

Partly

Is the work clearly and accurately presented and does it cite the current literature?

Yes

If applicable, is the statistical analysis and its interpretation appropriate?

Yes

Are all the source data underlying the results available to ensure full reproducibility?

Partly

Are the conclusions drawn adequately supported by the results?

Partly

Are sufficient details of methods and analysis provided to allow replication by others?

No

Reviewer Expertise:

Urban soil and plant selection. Site assessment and soil modification

I confirm that I have read this submission and believe that I have an appropriate level of expertise to confirm that it is of an acceptable scientific standard, however I have significant reservations, as outlined above.

# Reviewer report: Reviewer response for version 1

**DOI:** 10.21956/openreseurope.14572.r26816

**Published:** 2021-06-04

**Authors:** Yiyun Chen

**Affiliations:** 1 School of Resource and Environmental Sciences, Wuhan University, Wuhan, China

The manuscript entitled "Spatial variability of urban forest topsoil properties towards representative and robust sampling design" aims to optimize the number of urban forest topsoil samples, and proposes the use of EC testing as a baseline measure that determines spatial variation in the topsoil. The manuscript is clearly written and is of novelty. 

- In the introduction, what's the definition of "regular spatial distributions"?
- It would be good to mention any assumptions required to use the formula by Brinton *et al.* (2019).
- The authors mentioned that "Soil variability characteristics in urban environments do not follow normal statistical distributions". So what's the impact of data distribution on the results and conclusions?

Is the study design appropriate and does the work have academic merit?

Yes

Is the work clearly and accurately presented and does it cite the current literature?

Yes

If applicable, is the statistical analysis and its interpretation appropriate?

Yes

Are all the source data underlying the results available to ensure full reproducibility?

Yes

Are the conclusions drawn adequately supported by the results?

Yes

Are sufficient details of methods and analysis provided to allow replication by others?

Yes

Reviewer Expertise:

Pedometrics

I confirm that I have read this submission and believe that I have an appropriate level of expertise to confirm that it is of an acceptable scientific standard.
